# Differences in guideline-recommended heart failure medication between Dutch heart failure clinics: an analysis of the CHECK-HF registry

**DOI:** 10.1007/s12471-020-01421-1

**Published:** 2020-05-19

**Authors:** G. C. M. Linssen, J. F. Veenis, H. P. Brunner-La Rocca, P. E. J. van Pol, D. J. M. Engelen, R. M. van Tooren, H. J. J. Koornstra-Wortel, A. W. Hoes, J. J. Brugts, CHECK-HF investigators

**Affiliations:** 1 Department of Cardiology, Hospital Group Twente, Almelo and Hengelo, Almelo, The Netherlands; 2 Erasmus Medical Center, Department of Cardiology, University Medical Center Rotterdam, Rotterdam, The Netherlands; 3 Department of Cardiology, Maastricht University Medical Centre, Maastricht, The Netherlands; 4 Department of Cardiology, Alrijne Ziekenhuis, Leiderdorp, The Netherlands; 5 CONNECT-HF Program, Netherlands Society of Cardiology, Utrecht, The Netherlands; 6 Department of Cardiology, Diakonessenhuis, Utrecht, The Netherlands; 7 Department of Cardiology, St. Antonius ziekenhuis, Nieuwegein, The Netherlands; 8 Department of Cardiology, Maasziekenhuis Pantein, Beugen (Boxmeer), The Netherlands; 9 Julius Center for Health Sciences and Primary Care, University Medical Center Utrecht and Utrecht University, Utrecht, The Netherlands

**Keywords:** Heart failure, HFrEF, HFmrEF, Guidelines, Adherence, Medication

## Abstract

**Background:**

Heart failure (HF) is associated with poor prognosis, high morbidity and mortality. The prognosis can be optimised by guideline adherence, which also can be used as a benchmark of quality of care. The purpose of this study was to evaluate differences in use of HF medication between Dutch HF clinics.

**Methods:**

The current analysis was part of a cross-sectional registry of 10,910 chronic HF patients at 34 Dutch outpatient clinics in the period of 2013 until 2016 (CHECK-HF), and focused on the differences in prescription rates between the participating clinics in patients with heart failure with reduced ejection fraction (HFrEF).

**Results:**

A total of 8,360 HFrEF patients were included with a mean age of 72.3 ± 11.8 years (ranging between 69.1 ± 11.9 and 76.6 ± 10.0 between the clinics), 63.9% were men (ranging between 54.3 and 78.1%), 27.3% were in New York Heart Association (NYHA) class III/IV (ranging between 8.8 and 62.1%) and the average estimated glomerular filtration rate (eGFR) was 59.6 ± 24.6 ml/min (ranging between 45.7 ± 23.5 and 97.1 ± 16.5).

The prescription rates ranged from 58.9–97.4% for beta blockers (*p* < 0.01), 61.9–97.1% for renin-angiotensin system (RAS) inhibitors (*p* < 0.01), 29.9–86.8% for mineralocorticoid receptor antagonists (MRAs) (*p* < 0.01), 0.0–31.3% for ivabradine (*p* < 0.01) and 64.9–100.0% for diuretics (*p* < 0.01). Also, the percentage of patients who received the target dose differed significantly, 5.9–29.1% for beta blockers (*p* < 0.01), 18.4–56.1% for RAS inhibitors (*p* < 0.01) and 13.2–60.6% for MRAs (*p* < 0.01).

**Conclusions:**

The prescription rates and prescribed dosages of guideline-recommended medication differed significantly between HF outpatient clinics in the Netherlands, not fully explained by differences in patient profiles.

**Electronic supplementary material:**

The online version of this article (10.1007/s12471-020-01421-1) contains supplementary material, which is available to authorized users.

## What’s new?

- In contemporary real-world practice, wide ranges of demography, severity of heart failure and comorbidities of HFrEF patients were observed between heart failure clinics in the Netherlands.
- The prescription rates and prescribed dosages of guideline-recommended heart failure medication differed significantly between centres, not fully explained by differences in patient profiles.
- In HFmrEF patients, overall use and doses of heart failure medication, and ranges between centres did not differ considerably from those in HFrEF.
- Practical recommendations to improve heart failure management in transmural networks are provided.

## Introduction

Heart failure (HF) is associated with a high symptom burden, morbidity and mortality [1–3]. Optimising guideline-recommended HF therapies improve health-related quality of life and prognosis [4–6]. However, in real-world practice, implementation and adherence to recommended treatment, a benchmark of quality of care, are suboptimal. A recent analysis of medication profiles of 22,476 unselected patients with a diagnosis of HF at hospital discharge between 2001 and 2015 derived from the Dutch PHARMO Database Network showed only partial improvement of prescribed HF medication over time [7]. The percentage of patients prescribed the combination of a beta blocker and an angiotensin-converting-enzyme (ACE) inhibitor or angiotensin receptor blocker increased from 24 to approximately 45% within this 15-year period. The percentage of patients who also used a mineralocorticoid receptor antagonist (MRA) reached approximately 20%. Notably, the probability of being prescribed these combinations decreased with increasing age and there was no significant increase in MRA prescriptions. Moreover, recent real-world registries demonstrated underuse of HF therapies despite clear evidence-based recommendations [8–10].

In fact, randomised clinical trials and surveys did not represent real-life HF populations [11–13]. Moreover, the distribution of recommended HF treatment and considerable practice variation between regions and hospitals are largely unexplained, but also unexplored.

In a large-scale real-world registry at Dutch HF outpatient clinics, we therefore investigated the differences in medical HF therapies and determinants of prescription of individual, recommended HF drugs in HFrEF patients [14, 15] among 34 HF clinics in the Netherlands.

## Methods

**Fig. 1:**
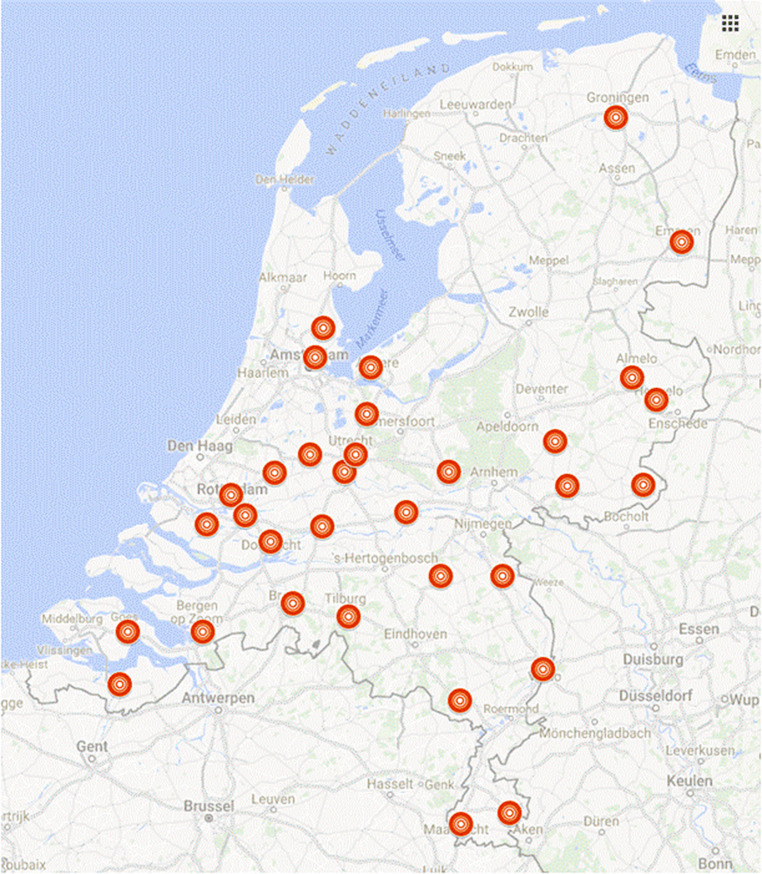
Geographical distribution of the 34 participating clinics of the CHECK-HF registry in the Netherlands

The design and methods of the CHECK-HF (*C*hronic *H*eart failure *E*SC guideline-based *C*ardiology practice Quality project) registry have been published in detail earlier [14]. Briefly, the CHECK-HF registry consists of 10,910 patients with chronic HF from a total of 34 participating centres (40% of the 86 centres in the Netherlands of which 60 have an outpatient HF unit) (Fig. 1). Patients were included cross-sectionally based on the available records of these patients. Between 2013 and 2016, all participating centres included patients diagnosed with HF based on the 2012 ESC guidelines on HF (i.e. based on symptoms and echo parameters) who were seen at the outpatient HF clinic (96%) or general cardiology outpatient clinic (4%) if no specific HF clinic was present.

Baseline patient characteristics, aetiology of HF, comorbidities, basic echocardiographic and electrocardiographic (ECG) parameters, laboratory markers, pacemaker, implantable cardioverter-defibrillator treatment and cardiac resynchronisation therapy as well as prescription rates of medication (drug name, dosage and frequency and total daily dose) were recorded. The target doses of guideline-recommended HF medication are presented in Suppl. Table 1. Drug doses were calculated compared with the recommended dose and according to guidelines as a daily dose or %, percentage of actual recommended daily dose.

Furthermore, contraindications and intolerance as indicated by the treating physician were collected. No predefined rules were applied to determine absolute contraindications.

In 283 (2.6%) patients, recording of ejection fraction in the database was insufficient to classify patients, so these patients were excluded from this analysis.

Based on echocardiographic results, the remaining 10,627 patients were divided based on left ventricular ejection fraction (LVEF) or visual assessment of the function of the left ventricle into HF with preserved ejection fraction (HFpEF) (LVEF ≥50%, *n* = 2,267 (21%)) and HF with reduced ejection fraction (HFrEF: LVEF <50%, *n* = 8,360 (79%)), according to the 2012 ESC HF guidelines [4].

For a sub-analysis according to the newer 2016 ESC HF guidelines, patients with an assessed LVEF <50% were categorised into HF with mid-range ejection fraction (HFmrEF) (LVEF 40–49%, *n* = 1,574 (19%)), HFrEF (LVEF <40%, *n* = 5,701 (68%)), and into HF with a semi-quantitative analysis of the systolic left ventricular function only (*n* = 1,085 (13%)). In the current analyses, we focused on the prescribed HF medication in HFrEF patients (LVEF <50%).

The Medical Research Ethics Committee of the Maastricht University Medical Center, the Netherlands, provided ethical approval for anonymously analysing existing patient data. No informed consent of the participants in this registry was required.

### Statistics

Continuous data are expressed as mean value ± standard deviation (SD) or median and interquartile range, depending on the distribution of the data, and compared by applying one-way analysis of variances (ANOVA) or Mann-Whitney U test as appropriate. Categorical data are expressed as counts and percentages, and compared by the Pearson chi-squared test. A two-sided *p*-value of 0.05 was considered statistically significant. Multivariable predictors for the use of HF medication associated with the hospital-ranked prescription of HF medication (beta blocker, renin-angiotensin system [RAS] inhibitor, MRA, ivabradine and diuretics, respectively) were sought, using multivariable logistic regression analysis, using the stepwise forward procedure. All predictors of medication use in univariable analysis at a *p*-value of <0.10 were included in the multivariable regression analysis. Results of logistic regression are presented as odds ratios (ORs) and confidence intervals (CIs).

All analyses were performed with SPSS Statistical Package version 25.0 (SPSS Inc, Chicago, Illinois).

## Results

Baseline characteristics of the total group of 8,360 HFrEF patients are shown in Table 1. Mean age was 72.3 ± 11.8 years (ranging between 69.1 ± 11.9 and 76.6 ± 10.0 between the clinics), 63.9% were men (ranging between 54.3 and 78.1%), 27.3% were in New York Heart Association (NYHA) class III/IV (ranging between 8.8 and 62.1%) and the average estimated glomerular filtration rate (eGFR) was 59.6 ± 24.6 ml/min (ranging between 45.7 ± 23.5 and 97.1 ± 16.5). Between centres, a wide range of prevalence rates with regard to ischaemic aetiology of HF, atrial fibrillation and comorbidities were found, as presented in Table 1. When subdividing HF patients in LVEF groups according to ESC guidelines 2016, HFmrEF patients (*n* = 1,574) were more often female, had less often ischaemic aetiology, less wide QRS complex and more often atrial fibrillation, hypertension and chronic obstructive pulmonary disease (COPD), all compared with HFrEF patients (*n* = 5,701). However, in both groups, there was a wide variation of all baseline characteristics between centres (Suppl. Tables 2 and 3).

**Table 1:** Baseline characteristics of HFrEF patients (LVEF <50%) and range between centres

	Overall population	Range
Number of patients	8,360	32; 1,549
Age (years) (*n* = 8,351)	72.27 ± 11.8	69.1 ± 11.9; 76.6 ± 10.0
Male gender (*n* = 8,323)	5,320 (63.9)	54.3; 78.1
BMI, kg/m2 (*n* = 7,671)	27.2 ± 5.2	26.2 ± 4.7; 28.4 ± 5.1
*NYHA* (*n* = 8,262)		
– I	1,313 (15.9)	0.0; 45.5
– II	4,692 (56.8)	35.0; 88.1
– III	2,108 (25.5)	8.8; 60.0
– IV	149 (1.8)	0.0; 9.6
LVEF, % (*n* = 6,179)	32.6 ± 10.5	28.4 ± 10.5; 44.2 ± 16.0
*Cause of HF* (*n* = 8,094)		
– Ischaemic cause of HF	4,182 (51.7)	34.9; 63.4
– Non-ischaemic cause of HF	3,912 (48.3)	36.6; 65.1
Systolic BP, mm Hg (*n* = 8,246)	125.7 ± 20.7	113.8 ± 19.6; 135.4 ± 22.7
Diastolic BP, mm Hg (*n* = 8,252)	71.2 ± 11.4	64.9 ± 10.4; 75.1 ± 12.9
Heart rate, bpm (*n* = 8,248)	72.0 ± 13.9	64.7 ± 8.0; 76.7 ± 17.1
Atrial fibrillation (*n* = 8,253)	2,109 (25.6)	12.2; 50.0
LBBB (*n* = 8,360)	1,414 (16.9)	0.0; 30.2
QRS ≥130 ms (*n* = 6,936)	2,774 (40.0)	0.0; 53.5
eGFR (*n* = 5,883)	59.6 ± 24.6	45.7 ± 23.5; 97.1 ± 16.5
*eGFR* (*n* = 5,883)		
– <30	667 (11.3)	0.0; 27.3
– 30–59	2,442 (41.5)	0.0; 54.5
– ≥60	2,774 (47.2)	18.2; 100.0
*Comorbidity* (*n* = 7,488)		
– Hypertension	2,978 (39.8)	7.8; 75.5
– Diabetes Mellitus	2,174 (29.0)	16.7; 51.0
– COPD	1,381 (18.4)	9.5; 29.9
– OSAS	495 (6.6)	0.0; 14.1
– Thyroid disease	557 (7.4)	0.6; 11.8
– Renal insufficiency^a^	3,950 (56.3)	30.5; 78.9
– No relevant comorbidity	855 (13.6)	0.0; 28.3

^a^Defined as eGFR <60 ml/min or a history of renal failure

*BMI* body mass index, *NYHA* New York Heart Association classification, *LVEF* left ventricular ejection fraction, *HF* heart failure, *HFrEF* HF with reduced ejection fraction, *HFmrEF* HF with mid-range ejection fraction, *HFpEF* HF with preserved ejection fraction, *BP* blood pressure, *LBBB* left bundle branch block, *eGFR* estimated glomerular filtration rate, *NT-proBNP N*-terminal pro-brain natriuretic peptide, *COPD* chronic obstructive pulmonary disease, *OSAS* obstructive sleep apnoea syndrome

### Guideline-recommended medical therapy in HFrEF

**Fig. 2:**
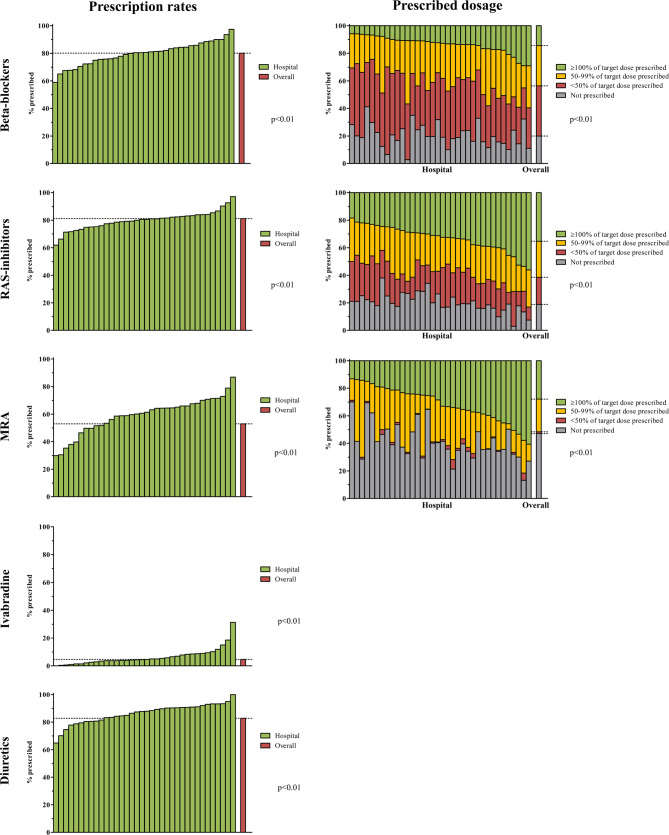
Prescription rates and prescribed dosages of HF medication in HFrEF patients (LVEF <50%) per participating clinic (*n* = 34) (*The left panels* show the order of hospitals on the x‑axis based on the percentage of prescription rate of each drug. *The red bar* is the overall presciption rate (%) and *the green bars* are the prescription rates (%) in each clinic. The same order is shown in *the panels on the right*.) (*HF* heart failure, *HFrEF* heart failure with reduced ejection fraction, *LVEF* left ventricular ejection fraction, *RAS* renin-angiotensin system, *MRA* mineralocorticoid receptor antagonists)

The prescription rates ranged between centres from 58.9–97.4% for beta blocker according to ESC guidelines 2012 (*p* < 0.01), 61.9–97.1% for renin-angiotensin system (RAS) inhibitors (*p* < 0.01), 29.9–86.8% for MRA (*p* < 0.01), 0.0–31.3% for ivabradine (*p* < 0.01) and 64.9–100.0% for diuretics (*p* < 0.01), see Table 2 and Fig. 2. In symptomatic HF patients (NYHA class II–IV), guideline-recommended medication only slightly differed from the total HFrEF group (Suppl. Table 4).

**Table 2:** Prescription rates of HF medication according to ESC Guidelines 2012 versus 2016 per participating clinic (*n* = 34)

		Guideline-recommended pharmacotherapy (average % (min.–max.))				
		Beta blocker	RAS inhibitor	MRA	Ivabradine	Diuretics
ESC Guidelines 2012	HFrEF	80.1 (58.9–97.4)	81.2 (61.9–97.1)	53.0 (29.9–86.8)	4.6 (0.0–31.3)	82.8(64.9–100.0)
ESC Guidelines 2016	HFrEF	81.0 (63.6–96.0)	83.2 (65.3–97.4)	56.4 (34.1–88.0)	5.4 (0.0–31.0)	83.4 (65.4–100.0)
	HFmrEF	77.7 (30.8–100.0)	76.8 (33.3–100.0)	45.1 (22.2–100.0)	3.1 (0.0–33.3)	79.5 (58.3–100.0)
	HFsemiq	78.6 (0.0–100.0)	77.6 (0.0–100.0)	46.3(0.0–100.0)	2.5 (0.0–30.8)	84.8 (0.0–100.0)

*HF* heart failure, *HFrEF* HF with reduced ejection fraction, *HFmrEF* HF with mid-range ejection fraction, *HFsemiq* HF with semiquantitatively estimated left ventricular ejection fraction—though <50%, *ESC* European Society of Cardiology, *RAS* renin-angiotensin system, *MRA* mineralocorticoid receptor antagonists

Dual therapy (beta blocker and RAS inhibitor) was prescribed in average 66.3% (min. 47.7 to max. 80.5) of HFrEF patients, one out of two in 28.7% (15.6–43.7) and none in 5.0% (0.9–13.5) respectively. Triple therapy (beta blocker, RAS inhibitor and MRA) was prescribed in average 35.6% (16.1–68.4) of HFrEF patients, two out of three in 45.7% (28.9–58.9), one out of three in 16.1% (0.0–24.7) and none in 2.6% (0.0–6.9) respectively. Also, the percentage of patients who received the target dose differed significantly, 5.9–29.1% for beta blocker (*p* < 0.01), 18.4–56.1% for RAS inhibitor (*p* < 0.01) and 13.2–60.6% for MRA (*p* < 0.01).

HFrEF patients seen at HF clinics received more often beta blockers, MRA, ivabradine and diuretics in comparison with those seen in general cardiology outpatient clinics, although rates of prescribed of RAS inhibitors were similar (Suppl. Table 5). Women with HFrEF less often received RAS inhibitors (79% vs 83%), but more often beta blockers (82% vs 79%) as compared with men. MRA were given in 53% of patients, both men and women (Suppl. Table 6).

Multivariable analysis of hospitals showed that the differences in prescribed HF medication between centres cannot be explained by clinical variables (Table 3, see Suppl. Table 7 for univariable analysis).

**Table 3:** Multivariable analysis of hospital differences in medical treatment of HFrEF patients (LVEF <50%)

		Beta blocker	RAS inhibitor	MRA	Ivabradine	Diuretics
		OR [95% CI]	OR [95% CI]	OR [95% CI]	OR [95% CI]	OR [95% CI]
Univariable	Hospital	1.05 [1.04–1.05]	1.04 [1.04–1.04]	1.06 [1.06–1.06]	1.09 [1.08–1.10]	1.06 [1.06–1.06]
Multivariable	Hospital	1.05 [1.04–1.06]	1.05 [1.04–1.06]	1.06 [1.05–1.07]	1.09 [1.07–1.10]	1.04 [1.03–1.05]
	Gender	1.20 [1.02–1.40]	–	–	–	1.31 [1.06–1.61]
	Age (per 10 years)	0.83 [0.78–0.89]	0.79 [0.72–0.87]	0.87 [0.83–0.91]	0.61 [0.56–0.67]	1.14 [1.04–1.25]
	BMI	–	1.04 [1.02–1.06]	1.02 [1.01–1.03]	–	1.06 [1.04–1.08]
	Systolic BP (per 10 mm Hg)	–	–	0.84 [0.82–0.87]	–	0.93 [0.87–1.00]
	Diastolic BP (per 10 mm Hg)	–	–	–	0.88 [0.79–0.98]	0.89 [0.80–1.00]
	NYHA classification	–	0.72 [0.63–0.82]	1.17 [1.08–1.27]	1.26 [1.05–1.50]	1.53 [1.30–1.80]
	Heart rate (per 10 beats/min)	–	0.84 [0.79–0.89]	–	–	1.12 [1.04–1.21]
	QRS duration (per 10 ms)	–	0.97 [0.95–0.99]	1.04 [1.02–1.05]	–	1.32 [1.01–1.72]
	eGFR (per 10 ml/min)	–	1.06 [1.01–1.11]	–	–	
	Ischaemic aetiology	–	0.76 [0.60–0.97]	–	–	
	Hypertension	1.22 [1.05–1.42]	–	–	–	
	Diabetes mellitus II	–	–	–	1.58 [1.21–2.08]	1.42 [1.11–1.81]
	COPD	–	–	–	1.58 [1.21–2.08]	1.32 [1.01–1.72]
	Renal insufficiency^a^	–	–	–	–	2.50 [2.03–3.09]

– variable not included in the model

*LVEF* left ventricular ejection fraction, *HF* heart failure, *HFrEF* HF with reduced ejection fraction, *OR* odds ratio, *CI* confidence interval, *RAS* renin-angiotensin system, *MRA* mineralocorticoid receptor antagonists, *BMI* body mass index, *NYHA* New York Heart Association, *BP* blood pressure, *eGFR* estimated glomerular filtration rate, *COPD* chronic obstructive pulmonary disease

^a^Defined as eGFR <60 ml/min or a history of renal failure

According to ESC guidelines 2016, the prescription rates in HF patients with LVEF <40%, both overall and ranges between centres of prescription rates of HF medication, were not different in a clinically meaningful way from HF with LVEF <50%.

### Medical treatment of HFmrEF and semi-quantitative patients

The distribution of beta blockers, RAS inhibitors and MRA in HFmrEF and semi-quantitative patients are shown in Table 2. Both overall prescription rates and ranges between centres did not differ in a clinically meaningful way from those in HFrEF patients. Also, in all LVEF groups, there was a wide range of prescribed dosages of HF medication percentages between centres (Suppl. Fig. 1, 2 and 3).

## Discussion

From our outpatient HF registry in a representative number of centres in the Netherlands, we demonstrated that demography, HF characteristics and comorbidities in HFrEF patients widely varied between those centres. Also, the prescription rates and prescribed dosages of guideline-recommended HF medication varied significantly, both for HFrEF and HFmrEF patients. Those variations between hospitals could not be explained by differences in baseline characteristics of participating HF patients.

Overall, we found higher prescription rates of recommended HF medication than in previous registries, which may be related to the delivery of specialist outpatient HF care in the vast majority of patients [10].

### Variation in prescribed heart failure medication

Remarkably, a wide distribution of prescribed medication between centres was observed. Many factors may play a role both in suboptimal therapy in the HF patients and in substantial variations between centres. Previously we reported from CHECK-HF that lower rates of guideline-directed pharmacotherapy in HFrEF patients were associated with increasing age, but much less influenced by comorbidities [10]. Recorded contraindications and intolerabilities did not explain the underuse of RAS inhibitors, beta blockers and MRA. Further analyses demonstrated that elderly heart failure patients with reduced ejection fraction (≥75 years) were prescribed significantly fewer beta blockers (77.8% vs 84.2%), RAS inhibitors (75.2% vs 89.7%), MRAs (50.6% vs 59.6%) and ivabradine (2.9% vs 9.3%), but significantly more diuretics (88.1% vs 72.6%) compared with patients aged less than 60 (*P* for all trends <0.01) [16]. In addition, the prescribed target dosages were significantly lower in elderly patients. Notably, patients with HFmrEF showed a similar trend in use of medication as in patients with HFrEF.

Also, recently reported data from the CHAMP-HF registry with 3,518 participating patients from 150 primary care and cardiology practices, demonstrated that lower medication utilisation or dose, was associated with older age, lower blood pressure, more severe functional class, renal insufficiency, and recent HF hospitalisation [9].

Notably, only 40% of the total HFrEF cohort of the Swedish Heart Failure Registry (11,215 patients, 27% women; mean age 75 ± 11 years) received an MRA [17]. Underuse of MRA was not related to hyperkalaemia, but it was, among other factors, related to impaired renal function (even moderately impaired), which is not a contraindication for MRA use. An explanation for the underuse of MRA might be the reluctance of prescribing an MRA to a vulnerable group of HF patients, already treated with an RAS inhibitor, beta blocker and in the majority of cases also a diuretic [18, 19]. Remarkably, age of patients in the present analysis had no impact on the differences in prescription of HF medication between centres.

Therefore, perceived polypharmacy, presence of comorbidities and overestimation of side-effects may influence use and dosing of evidence-based medication. In addition, patient preferences and family caregiver perceptions may influence therapeutic decisions [20]. Furthermore, an analysis by the BIOSTAT-CHF study group suggested that women with HFrEF might need lower doses of RAS inhibitors and beta blockers than men, also adjusted for age [21].

However, it is unclear why not only new medication, e.g. ivabradine and more recently sacubitril/valsartan, but also long-standing, established, disease-modifying therapies are not widely adopted nor fully prescribed. Therefore, it is important to gain detailed insights in reasons for not adopting recommended therapies both at a hospital level and at an individual patient level. Assessing information on real motivation of medical decisions and perceived barriers would contribute to effective improvement of HF care.

Importantly, suboptimal use of HF medication may have detrimental effects on clinical outcomes. Adherence to guideline-directed therapy of HFrEF, with prescription of at least 50% of the target dosage is associated with better outcome [6, 22], at least in younger patients with little comorbidities [23].

### Optimising heart failure management

Although nonadherence to guideline-directed HF therapies is not fully understood, several practical recommendations to improve HF management can be made (Suppl. Table 8).

Obviously, being informed on performance of health care professionals involved in HF management, will contribute to improving delivery of care. Therefore, the CHECK-HF centres received individual feedback and in national meetings possible solutions to optimise HF care were shared. Furthermore, a nationwide, structured HF registry is being launched.

Acknowledging that HF care should be delivered seamless to patients, the Netherlands Society of Cardiology, started the CONNECT Heart Failure programme, in which concepts of integrated collaboration were translated towards detailed protocols by joint health care professionals in geographic regions [24]. These collaborations also provide strategies for optimising diagnostic pathways and HF therapies, accompanied by educational activities for professional teams. The initiated national registry will provide information on the effectiveness of incorporating these strategies.

At a patient level, clinical judgment of the heart failure syndrome, management of comorbidities, in concert with optimally implemented disease-modifying therapies are of pivotal importance [25–27]. In addition, blood pressure, renal function and hyperkalaemia may limit up-titration of all recommended drugs [28]. This may be even more complicated by the fact that the number of drug classes shown to improve outcome in HFrEF is increasing [29]. Among potential solutions are start-low and go-slow dosing strategies, close monitoring of vital parameters and side-effects, the use of new potassium binders and angiotensin receptor/neprilysin inhibition. Critical appraisal and reduction of co-medication may also be beneficial. In addition, pharmacy care improves adherence to HF medications and quality of life, which was recently demonstrated by the PHARM-CHF investigators [30].

In concert with dedicated efforts of professional HF teams, well-informed patients and family caregivers may empower their participation in medical decision-making and contributes to earlier access of new therapies [5, 24]. Informed treatment choices are of particular relevance in guidance of decisions during advanced and palliative stages of care.

## Limitations and strenghts

The CHECK-HF registry is a large-scale real-world registry of HF outpatient clinics in the Netherlands reflective of Western European countries. However, some limitations should be mentioned, such as the cross-sectional design limiting follow-up data on patient outcomes. Some missing data exists, which might influence results. Our registry included only patients seen in secondary, but not in primary care, which limits the generalisability of our findings to the primary care setting. Information on actual protocols of diagnostic workup and medical decision-making strategies in centres was not collected. Notably, the CHECK-HF inclusion period was from 2013 till end of 2016, in which the CONNECT programme for Heart failure regional care had been in the initial phase of implementation in regions. Therefore, we have not collected data on adoption of the CONNECT Heart Failure programme in the centres. Strengths of the study are the reflection of the true practice of large scale nationwide outpatient HF management with detailed information on medication prescription and dosage.

## Conclusion

In this Dutch real-world registry of outpatient HF population, wide between-clinic ranges of demography, severity of heart failure and comorbidities of HF patients were observed. Also the prescription rates and prescribed dosages of guideline-recommended HF medication differed significantly, not fully explained by differences in the patient profiles. Thus, future research should lead to strategies to improve management of HF patients including reduction of practice variation.

## Caption Electronic Supplementary Material

**1. Suppl. Table 1.** Target daily doses of guideline-recommended therapy in patients with HFrEF

**2. Suppl. Table 2.** Baseline characteristics in HFrEF patients (LVEF <40%) and range between centres

**3. Suppl. Table 3.** Baseline characteristics in HFmrEF patients (LVEF 40–49%) and range between centres

**4. Suppl. Table 4.** Prescription rates of HF medication according to ESC Guidelines 2012 versus 2016 per participating clinic (*n* = 34), only for symptomatic patients

**5. Suppl. Table 5.** Prescription rates of HF medication according to ESC Guidelines 2012 versus 2016 per participating clinic (*n* = 34)

**6. Suppl. Table 6.** Prescription rates of HF medication according to ESC Guidelines 2012 versus 2016 per participating clinic (*n* = 34)

**7. Suppl. Table 7**. Univariable analysis of predictors of HF medical treatment of HFrEF patients (LVEF <50%), Odds Ratios [95% confidence intervals]

**8. Suppl. Table 8.** Practical recommendations for optimal use of guideline-directed heart failure therapies

**9. Suppl. Fig. 1. **Prescription rates (%) and prescribed dosages (%) of HF medication in HFrEF patients (LVEF <40%) per participating clinic (*n* = 34) (*The left panels* show the order of hospitals on the x‑axis based on the percentage of prescription rate of each drug. *The red bar* is the overall prescription rate (%) and *the green bars* are the prescription rates (%) in each clinic. The same order is shown in *the panels on the right*). (*HF* heart failure, *HFrEF* heart failure with reduced ejection fraction, *LVEF* left ventricular ejection fraction, *RAS* renin-angiotensin system, *MRA* mineralocorticoid receptor antagonists)

**10. Suppl. Fig. 2**. Prescription rates (%) and prescribed dosages (%) of HF medication in HFmrEF patients (LVEF 40–49%) per participating clinic (*n* = 34) (*The left panels* show the order of hospitals on the x‑axis based on the percentage of prescription rate of each drug. *The red bar* is the overall prescription rate (%) and *the green bars* are the prescription rates (%) in each clinic. The same order is shown in *the panels on the right*). (*HF* heart failure, *HFmrEF* heart failure with mid-range ejection fraction, *LVEF* left ventricular ejection fraction, *RAS* renin-angiotensin system, *MRA* mineralocorticoid receptor antagonists)

**11. Suppl. Fig. 3**. Prescription rates and prescribed dosages of HF medication in HF patients with semiquantitatively measured LV function per participating clinic (*n* = 27) (*The left panels* show the order of hospitals on the x‑axis based on the percentage of prescription rate of each drug. *The red bar* is the overall prescription rate (%) and *the green bars* are the prescription rates (%) in each clinic. The same order is shown in *the panels on the right*). (*HF* heart failure, *LV* left ventricular, *RAS* renin-angiotensin system, *MRA* mineralocorticoid receptor antagonists)

## References

[1] Metra M, Teerlink JR (2017). Heart failure. Lancet.

[2] Koudstaal S, Pujades-Rodriguez M, Denaxas S (2017). Prognostic burden of heart failure recorded in primary care, acute hospital admissions, or both: a population-based linked electronic health record cohort study in 2.1 million people. Eur J Heart Fail.

[3] van Deursen VM, Urso R, Laroche C (2014). Co-morbidities in patients with heart failure: an analysis of the European Heart Failure Pilot Survey. Eur J Heart Fail.

[4] McMurray JJ, Adamopoulos S, Anker SD (2012). ESC Guidelines for the diagnosis and treatment of acute and chronic heart failure 2012: The Task Force for the Diagnosis and Treatment of Acute and Chronic Heart Failure 2012 of the European Society of Cardiology. Developed in collaboration with the Heart Failure Association (HFA) of the ESC. Eur Heart J.

[5] Ponikowski P, Voors AA, Anker SD (2016). 2016 ESC Guidelines for the diagnosis and treatment of acute and chronic heart failure: The Task Force for the diagnosis and treatment of acute and chronic heart failure of the European Society of Cardiology (ESC). Developed with the special contribution of the Heart Failure Association (HFA) of the ESC. Eur J Heart Fail.

[6] Komajda M, Schöpe J, Wagenfleil S (2019). Physicians’ guideline adherence is associated with long-term heart failure mortality in outpatients with heart failure with reduced ejection fraction: the QUALIFY international registry. Eur J Heart Fail.

[7] Kruik-Kollöffel WJ, Linssen GCM, Kruik HJ (2019). Effects of European Society of Cardiology guidelines on medication profiles after hospitalization for heart failure in 22,476 Dutch patients: from 2001 until 2015. Heart Fail Rev.

[8] Crespo-Leiro MG, Anker SD, Maggioni AP (2016). European Society of Cardiology Heart Failure Long-Term Registry (ESC-HF-LT): 1-year follow-up outcomes and differences across regions. Eur J Heart Fail.

[9] Greene SJ, Butler J, Albert NM (2018). Medical therapy for heart failure with reduced ejection fraction: The CHAMP-HF Registry. J Am Coll Cardiol.

[10] Brunner-La Rocca HP, Linssen GC, Smeele FJ (2019). Contemporary drug treatment of chronic heart failure with reduced ejection fraction. The CHECK-HF registry. J Am Coll Cardiol.

[11] Flather MD, Shibata MC, Coats AJ (2005). Randomized trial to determine the effect of nebivolol on mortality and cardiovascular hospital admission in elderly patients with heart failure (SENIORS). Eur Heart J.

[12] Van Spall HG, Toren A, Kiss A, Fowler RA (2007). Eligibility criteria of randomized controlled trials published in high-impact general medical journals: a systematic sampling review. JAMA.

[13] Burnett H, Earley A, Voors AA (2017). Thirty years of evidence on the efficacy of drug treatments for chronic heart failure with reduced ejection fraction: a network meta-analysis. Circ Heart Fail.

[14] Brugts JJ, Linssen GCM, Hoes AW, Brunner-La Rocca HP, Investigators of CHECK-HF (2018). Real-world heart failure management in 10,910 patients with chronic heart failure in the Netherlands: design and rationale of the Chronic Heart failure ESC guideline-based Cardiology practice Quality project (CHECK-HF) registry. Neth Heart J.

[15] Brunner-La Rocca HP, Linssen GC, Smeele FJ (2019). Contemporary drug treatment of chronic heart failure with reduced ejection fraction. The CHECK-HF registry. J Am Coll Cardiol.

[16] Veenis JF, Brunner-La Rocca HP, Linssen GC (2019). CHECK-HF investigators. Age differences in contemporary treatment of patients with chronic heart failure and reduced ejection fraction. Eur J Prev Cardiol.

[17] Savarese G, Carrero JJ, Pitt B (2018). Factors associated with underuse of mineralocorticoid receptor antagonists in heart failure with reduced ejection fraction: an analysis of 11 215 patients from the Swedish Heart Failure Registry. Eur J Heart Fail.

[18] Ferreira JP, Rossignol P, Machu JL (2017). Mineralocorticoid receptor antagonist pattern of use in heart failure with reduced ejection fraction: findings from BIOSTAT-CHF. Eur J Heart Fail.

[19] Savarese G, Dahlström U, Vasko P (2018). Association between renin-angiotensin system inhibitor use and mortality/morbidity in elderly patients with heart failure with reduced ejection fraction: a prospective propensity score-matched cohort study. Eur Heart J.

[20] Brunner-La Rocca HP, Rickenbacher P, Muzzarelli S (2012). End-of-life preferences of elderly patients with chronic heart failure. Eur Heart J.

[21] Santema BT, Ouwerkerk W, Tromp J (2019). Identifying optimal doses of heart failure medications in men compared with women: a prospective, observational, cohort study. Lancet.

[22] Ouwerkerk W, Voors AA, Anker SD (2017). Determinants and clinical outcome of uptitration of ACE-inhibitors and beta-blockers in patients with heart failure: a prospective European study. Eur Heart J.

[23] Brunner-La Rocca HP, Eurlings L, Richards AM (2015). Which heart failure patients profit from natriuretic peptide guided therapy? A meta-analysis from individual patient data of randomized trials. Eur J Heart Fail.

[24] Lucas CMHB, van Pol PEJ, Eysink Smeets JBE (2015). Heart failure in 2015: let’s get organised!. Neth Heart J.

[25] Rossignol P, Hernandez AF, Solomon SD, Zannad F (2019). Heart failure drug treatment. Lancet.

[26] Ambrosy AP, Gheorghiade M (2017). Real-world dosing of evidence-based medications for heart failure: embracing guideline recommendations and clinical judgement. Eur J Heart Fail.

[27] Yancy CW, Januzzi JL, Allen LA (2018). 2017 ACC Expert Consensus decision pathway for optimization of heart failure treatment: answers to 10 pivotal issues about heart failure with reduced ejection fraction: a report of the American College of Cardiology Task Force on expert consensus decision pathways. J Am Coll Cardiol.

[28] Marti CN, Fonarow GC, Anker SD (2019). Medication dosing for heart failure with reduced ejection fraction—opportunities and challenges. Eur J Heart Fail.

[29] McMurray JJV, Solomon SD, Inzucchi SE (2019). Dapagliflozin in patients with heart failure and reduced ejection fraction. N Engl J Med.

[30] Schulz M, Griese-Mammen N, Anker SD (2019). Pharmacy-based interdisciplinary intervention for patients with chronic heart failure: results of the PHARM-CHF randomized controlled trial. Eur J Heart Fail.

