# Supplementary material for: Differences in guideline-recommended heart failure medication between Dutch heart failure clinics: an analysis of the CHECK-HF registry
Source: Neth Heart J. 2020 May 19;28(6):334–44. doi: 10.1007/s12471-020-01421-1 (PMC7270463; doi:10.1007/s12471-020-01421-1)
Supplement: Supplementary file 1 — 1. Suppl. Table 1. Target daily doses of guideline-recommended therapy in patients with HFrEF [file 12471_2020_1421_MOESM1_ESM.docx]

| **Suppl. Table 1.** Target daily doses of guideline-recommended therapy in patients with HFrEF | | |
| --- | --- | --- |
| **Beta blocker** | |  |
|  | Bisoprolol | 10 mg |
|  | Carvedilol | 50 mg |
|  | Metoprolol succinate | 200 mg |
|  | Nebivolol | 10 mg |
| **ACE inhibitor** | |  |
|  | Captopril | 150 mg |
|  | Enalapril | 20 mg |
|  | Lisinopril | 40 mg |
|  | Ramipril | 10 mg |
|  | Perindopril | 8 mg |
| **ARB** | |  |
|  | Candesartan | 32 mg |
|  | Losartan | 150 mg |
|  | Valsartan | 320 mg |
| **MRA** | |  |
|  | Eplerenone | 50 mg |
|  | Spironolactone | 25 mg |
| *ACE* angiotensin-converting enzyme, *ARB* angiotensin II receptor blocker, *MRA* mineralocorticoid receptor antagonist, *HFrEF* heart failure with reduced ejection fraction | | |
