# Supplementary material for: Differences in guideline-recommended heart failure medication between Dutch heart failure clinics: an analysis of the CHECK-HF registry
Source: Neth Heart J. 2020 May 19;28(6):334–44. doi: 10.1007/s12471-020-01421-1 (PMC7270463; doi:10.1007/s12471-020-01421-1)
Supplement: Supplementary file 2 — 2. Suppl. Table 2. Baseline characteristics in HFrEF patients (LVEF <40%) and range between centres [file 12471_2020_1421_MOESM2_ESM.docx]

| **Suppl. Table 2.** Baseline characteristics in HFrEF patients (LVEF<40%) and range between centres |
| --- |

|  |  | **Overall population** | **Range** |
| --- | --- | --- | --- |
| Number of patients | | 5,701 | 25; 785 |
| Age (years) (*n*=5,694) | | 71.4±11.8 | 66.9±11.8; 75.8±10.3 |
| Male gender (*n*=5,677) | | 3,767 (66.4) | 57.5; 82.9 |
| BMI, kg/m2 (*n*=5,276) | | 27.2±5.1 | 25.9±3.7; 29.1±6.9 |
| NYHA (*n*=5,643) | |  |  |
|  | I | 839 (14.9) | 0.0; 46.2 |
|  | II | 3,244 (57.5) | 33.3; 87.5 |
|  | III | 1,449 (25.7) | 7.7; 62.7 |
|  | IV | 111 (2.0) | 0.0; 8.9 |
| LVEF, % (*n*=4,880) | | 29.3±9.0 | 26.7±8.5; 38.1±16.5 |
| Cause of HF (*n*=5,505) | |  |  |
|  | Ischaemic cause of HF | 2,945 (53.5) | 35.1; 69.3 |
|  | Non-ischaemic cause of HF | 2,560 (46.5) | 30.7; 64.9 |
| Systolic BP, mmHg (*n*=5,613) | | 124.4±20.2 | 111.5±18.3; 133.6±22.7 |
| Diastolic BP, mmHg (*n*=5,615) | | 71.2±11.2 | 64.3±10.6; 75.8±12.9 |
| Heart rate, bpm (*n*=5,624) | | 71.9±13.8 | 64.8±8.0; 77.6±16.8 |
| Atrial fibrillation (*n*=5,625) | | 1,258 (22.4) | 12.2; 56.0 |
| LBBB (*n*=5,701) | | 1,050 (18.4) | 0.0; 32.4 |
| QRS ≥130 ms (*n*=4,824) | | 2,080 (43.1) | 0.0; 58.1 |
| eGFR (*n*=4,178) | | 61.2±24.9 | 48.5±26.2; 97.5±16.7 |
| eGFR (*n*=4,178) | |  |  |
|  | <30 | 430 (10.3) | 0.0; 19.5 |
|  | 30-59 | 1,676 (40.1) | 0.0; 63.6 |
|  | ≥60 | 2,072 (49.6) | 18.2; 100.0 |
| Comorbidity (*n*=5,073) | |  |  |
|  | Hypertension | 1,944 (38.3) | 5.6; 76.7 |
|  | Diabetes Mellitus | 1,481 (29.2) | 17.6; 51.4 |
|  | COPD | 900 (17.7) | 6.7; 33.3 |
|  | OSAS | 320 (6.3) | 0.0; 16.7 |
|  | Thyroid disease | 368 (7.3) | 0.0; 12.2 |
|  | Renal insufficiency † | 2,741 (54.8) | 28.6; 84.0 |
|  | No relevant comorbidity | 671 (15.0) | 0.0; 33.3 |
| † Defined as eGFR <60ml/min or a history of renal failure  *BMI* body mass index, *NYHA* New York Heart Association classification, *LVEF* left ventricular ejection fraction, *HF* heart failure, *HFrEF* HF with reduced ejection fraction; *BP* blood pressure, *LBBB* left bundle branch block, *eGFR* estimated glomerular filtration rate, *NT-proBNP* N-terminal pro-brain natriuretic peptide, *COPD* chronic obstructive pulmonary disease, *OSAS* obstructive sleep apnoea syndrome | | | |
