# Supplementary material for: Differences in guideline-recommended heart failure medication between Dutch heart failure clinics: an analysis of the CHECK-HF registry
Source: Neth Heart J. 2020 May 19;28(6):334–44. doi: 10.1007/s12471-020-01421-1 (PMC7270463; doi:10.1007/s12471-020-01421-1)
Supplement: Supplementary file 3 — 3. Suppl. Table 3. Baseline characteristics in HFmrEF patients (LVEF 40–49%) and range between centres [file 12471_2020_1421_MOESM3_ESM.docx]

| \| **Suppl. Table 3.** Baseline characteristics in HFmrEF patients (LVEF 40-49%) and range between centres \| \| \| \| \| --- \| --- \| --- \| --- \| \|  \|  \| **Overall population** \| **Range** \| \| Number of patients \| \| 1,574 \| 3; 415 \| \| Age (years) (*n*=1,573) \| \| 73.7±11.7 \| 68.1±12.8; 80.9±6.8 \| \| Male gender (*n*=1,571) \| \| 917 (58.4) \| 34.5; 100.0 \| \| BMI, kg/m2 (*n*=1,462) \| \| 27.5±5.4 \| 24.0±7.0; 30.5±4.4 \| \| NYHA (*n*=1,558) \| \|  \|  \| \|  \| I \| 284 (18.2) \| 0.0; 50.0 \| \|  \| II \| 854 (54.8) \| 22.2; 90.0 \| \|  \| III \| 392 (25.2) \| 5.5; 66.7 \| \|  \| IV \| 28 (1.8) \| 0.0; 11.6 \| \| LVEF, % (*n*=1,299) \| \| 45.0±5.4 \| 46.2±5.3; 48.8±9.0 \| \| Cause of HF (*n*=1,521) \| \|  \|  \| \|  \| Ischaemic cause of HF \| 691 (45.4) \| 11.1; 70.0 \| \|  \| Non-ischaemic cause of HF \| 830 (54.6) \| 30.0; 88.9 \| \| Systolic BP, mmHg (*n*=1,556) \| \| 129.5±21.6 \| 113.5±17.5; 138.7±21.9 \| \| Diastolic BP, mmHg (*n*=1,560) \| \| 71.8±12.0 \| 58.3±13.9; 77.2±8.6 \| \| Heart rate, bpm (*n*=1,554) \| \| 72.5±14.3 \| 63.2±10.0; 79.3±17.9 \| \| Atrial fibrillation (*n*=1,559) \| \| 534 (34.3) \| 9.5; 71.4 \| \| LBBB (*n*=1,574) \| \| 216 (13.7) \| 0.0; 66.7 \| \| QRS ≥130 ms (*n*=1,320) \| \| 416 (31.5) \| 0.0; 100.0 \| \| eGFR (*n*=973) \| \| 56.2±23.7 \| 35.8±3.0; 96.4±9.9 \| \| eGFR (*n*=973) \| \|  \|  \| \|  \| <30 \| 133 (13.7) \| 0.0; 45.5 \| \|  \| 30-59 \| 439 (45.1) \| 0.0; 100.0 \| \|  \| ≥60 \| 401 (41.2) \| 0.0; 100.0 \| \| Comorbidity (*n*=1,417) \| \|  \|  \| \|  \| Hypertension \| 619 (43.7) \| 0.0; 81.8 \| \|  \| Diabetes Mellitus \| 397 (28.0) \| 0.0; 66.7 \| \|  \| COPD \| 291 (20.5) \| 0.0; 44.4 \| \|  \| OSAS \| 116 (8.2) \| 0.0; 28.6 \| \|  \| Thyroid disease \| 111 (7.8) \| 0.0; 33.3 \| \|  \| Renal insufficiency † \| 745 (60.9) \| 0.0; 100.0 \| \|  \| No relevant comorbidity \| 98 (9.0) \| 0.0; 38.9 \| \|  \| \| \| \| \| † Defined as eGFR <60mL/min or a history of renal failure  *BMI* body mass index, *NYHA* New York Heart Association classification, *LVEF* left ventricular ejection fraction, *HF* heart failure, *HFmrEF* HF with mid-range ejection fraction; *BP* blood pressure, *LBBB* left bundle branch block, *eGFR* estimated glomerular filtration rate, *NT-proBNP* N-terminal pro-brain natriuretic peptide, *COPD* chronic obstructive pulmonary disease, *OSAS* obstructive sleep apnoea syndrome \| \| \| \| |
| --- | --- | --- | --- | --- | --- | --- | --- | --- | --- | --- | --- | --- | --- | --- | --- | --- | --- | --- | --- | --- | --- | --- | --- | --- | --- | --- | --- | --- | --- | --- | --- | --- | --- | --- | --- | --- | --- | --- | --- | --- | --- | --- | --- | --- | --- | --- | --- | --- | --- | --- | --- | --- | --- | --- | --- | --- | --- | --- | --- | --- | --- | --- | --- | --- | --- | --- | --- | --- | --- | --- | --- | --- | --- | --- | --- | --- | --- | --- | --- | --- | --- | --- | --- | --- | --- | --- | --- | --- | --- | --- | --- | --- | --- | --- | --- | --- | --- | --- | --- | --- | --- | --- | --- | --- | --- | --- | --- | --- | --- | --- | --- | --- | --- | --- | --- | --- | --- | --- | --- | --- | --- | --- | --- | --- | --- | --- | --- | --- | --- | --- | --- | --- | --- | --- | --- | --- | --- | --- | --- | --- | --- | --- | --- | --- |
