# Supplementary material for: Differences in guideline-recommended heart failure medication between Dutch heart failure clinics: an analysis of the CHECK-HF registry
Source: Neth Heart J. 2020 May 19;28(6):334–44. doi: 10.1007/s12471-020-01421-1 (PMC7270463; doi:10.1007/s12471-020-01421-1)
Supplement: Supplementary file 4 — 4. Suppl. Table 4. Prescription rates of HF medication according to ESC Guidelines 2012 versus 2016 per participating clinic (n = 34), only for symptomatic patients [file 12471_2020_1421_MOESM4_ESM.docx]

| **Suppl. Table 4.** Prescription rates of HF medication according to ESC Guidelines 2012 versus 2016 per participating clinic (*n*=34), only for symptomatic patients | | | | | | |
| --- | --- | --- | --- | --- | --- | --- |
|  |  | **Guideline-recommended pharmacotherapy (average % (min.-max.))** | | | | |
|  |  | **Beta blocker** | **RAS inhibitor** | **MRA** | **Ivabradine** | **Diuretics** |
| **ESC Guidelines 2012** | HFrEF | 80.2 (59.6-97.4) | 80.4 (50.0- 97.0) | 55.5 (30.6-86.8) | 5.0 (0.0-40.9) | 85.7 (65.9-100.0) |
|  |  |  |  |  |  |  |
| **ESC Guidelines**  **2016** | HFrEF | 81.3 (64.5-96.0) | 82.3 (54.0-97.3) | 58.1 (34.7-88.0) | 5.7 (0.0-40.0) | 85.5 (65.9-100.0) |
|  | HFmrEF | 78.2 (30.8-100.0) | 75.6 (33.3-100.0) | 49.1 (23.6-100.0) | 3.6 (0.0-50.0) | 84.4 (63.6-100.0) |
|  | HFsemiq | 77.3 (0.0-100.0) | 77.1 (33.3-100.0) | 50.2 (0.0-100.0) | 3.0 (0.0-30.8) | 88.8 (69.2-100.0) |
| *HF* heart failure; *HFrEF* HF with reduced ejection fraction, *HFmrEF* HF with mid-range ejection fraction,  *HFsemiq* HF with semiquantitatively estimated left ventricular ejection fraction - though <50%, *ESC* European Society of Cardiology,  *RAS* renin-angiotensin system, *MRA* mineralocorticoid receptor antagonists | | | | | | |
