# Supplementary material for: Differences in guideline-recommended heart failure medication between Dutch heart failure clinics: an analysis of the CHECK-HF registry
Source: Neth Heart J. 2020 May 19;28(6):334–44. doi: 10.1007/s12471-020-01421-1 (PMC7270463; doi:10.1007/s12471-020-01421-1)
Supplement: Supplementary file 5 — 5. Suppl. Table 5. Prescription rates of HF medication according to ESC Guidelines 2012 versus 2016 per participating clinic (n = 34) [file 12471_2020_1421_MOESM5_ESM.docx]

| **Suppl. Table 5.** Prescription rates of HF medication according to ESC Guidelines 2012 versus 2016 per participating clinic (*n*=34) | | | | | | | |
| --- | --- | --- | --- | --- | --- | --- | --- |
|  |  |  | **Guideline-recommended pharmacotherapy (*n* ( %))** | | | | |
|  |  |  | **Beta blocker** | **RAS inhibitor** | **MRA** | **Ivabradine** | **Diuretics** |
| **ESC Guidelines 2012** | HFrEF | Patients seen at HF clinic | 6,303 (80.6) | 6,358 (81.3) | 4,204 (53.7) | 382 (4.8) | 6,543 (83.7) |
|  |  | Patients not seen at HF clinic | 240 (68.0) | 288 (81.6) | 123 (34.8) | 2 (0.6) | 225 (63.7) |
|  |  | p-value | <0.01 | 0.88 | <0.01 | <0.01 | <0.01 |
|  |  |  |  |  |  |  |  |
| **ESC Guidelines**  **2016** | HFrEF | Patients seen at HF clinic | 4,320 (81.6) | 4,403 (83.2) | 3,040 (57.4) | 306 (5.7) | 4,468 (84.4) |
|  |  | Patients not seen at HF clinic | 205 (69.0) | 251 (84.5) | 108 (36.4) | 2 (0.7) | 191 (64.3) |
|  |  | p-value | <0.01 | 0.55 | <0.01 | <0.01 | <0.01 |
|  | HFmrEF | Patients seen at HF clinic | 1,151 (78.3) | 1,134 (77.1) | 674 (45.9) | 49 (3.2) | 1,178 (80.2) |
|  |  | Patients not seen at HF clinic | 32 (61.5) | 34 (65.4) | 13 (25.)0 | 0 (0.0) | 31 (59.6) |
|  |  | p-value | <0.01 | 0.05 | <0.01 | 0.41 | <0.01 |
|  | HFsemiq | Patients seen at HF clinic | 832 (78.6) | 821 (77.5) | 490 (46.3) | 27 (2.5) | 897 (84.8) |
|  |  | Patients not seen at HF clinic | 3 (75.0) | 3 (75.0) | 2 (50.0) | 0 (0.0) | 3 (75.0) |
|  |  | p-value | 1.00 | 1.00 | 1.00 | 1.00 | 0.49 |
| *HF* heart failure; *HFrEF* HF with reduced ejection fraction, *HFmrEF* HF with mid-range ejection fraction,  *HFsemiq* HF with semiquantitatively estimated left ventricular ejection fraction - though <50%, *ESC* European Society of Cardiology,  *RAS* renin-angiotensin system, *MRA* mineralocorticoid receptor antagonists | | | | | | | |
