# Supplementary material for: Differences in guideline-recommended heart failure medication between Dutch heart failure clinics: an analysis of the CHECK-HF registry
Source: Neth Heart J. 2020 May 19;28(6):334–44. doi: 10.1007/s12471-020-01421-1 (PMC7270463; doi:10.1007/s12471-020-01421-1)
Supplement: Supplementary file 6 — 6. Suppl. Table 6. Prescription rates of HF medication according to ESC Guidelines 2012 versus 2016 per participating clinic (n = 34) [file 12471_2020_1421_MOESM6_ESM.docx]

| **Suppl. Table 6.** Prescription rates of HF medication according to ESC Guidelines 2012 versus 2016 per participating clinic (*n*=34) | | | | | | | |
| --- | --- | --- | --- | --- | --- | --- | --- |
|  |  |  | **Guideline-recommended pharmacotherapy (*n* (%))** | | | | |
|  |  |  | **Beta blocker** | **RAS inhibitor** | **MRA** | **Ivabradine** | **Diuretics** |
| **ESC Guidelines 2012** | HFrEF | Men | 79.1 (54.9-96.1) | 82.6 (66.7- 97.6) | 52.8 (26.6-79.2) | 4.2 (0.0-14.3) | 81.6 (60.7-100.0) |
|  |  | Women | 82.0 (64.9-100.0) | 78.9 (54.8- 96.1) | 53.3 (26.4-100.0) | 5.2 (0.0-28.6) | 85.0 (73.2-100.0) |
|  |  |  |  |  |  |  |  |
| **ESC Guidelines**  **2016** | HFrEF | Men | 79.7 (61.7-95.7) | 84.2 (67.6- 97.7) | 55.7 (30.0-83.3) | 4.9 (0.0-31.8) | 82.3 (60.2-100.0) |
|  |  | Women | 83.7 (64.5-100.0) | 81.1 (57.1- 96.6) | 57.7 (33.0-100.0) | 6.3 (0.0-32.4) | 85.7 (65.9-100.0) |
|  | HFmrEF | Men | 76.9 (0.0-100.0) | 77.9 (33.3- 100.0) | 44.0 (11.8-100.0) | 3.1 (0.0-33.3) | 76.6 (58.8-100.0) |
|  |  | Women | 79.0 (47.1-100.0) | 75.2 (35.7- 100.0) | 46.9 (0.0-100.0) | 3.2 (0.0-17.6) | 83.4 (50.0-100.0) |
|  | HFsemiq | Men | 78.3 (0.0-100.0) | 79.4 (0.0- 100.0) | 47.9 (0.0-100.0) | 1.9 (0.0-28.6) | 84.5 (66.7-100.0) |
|  |  | Women | 79.0 (0.0-100.0) | 74.6 (0.0- 100.0) | 43.4 (0.0-100.0) | 3.4 (0.0-50.0) | 85.5 (0.0-100.0) |
| ____________________________________________________________________________________________________  *HF* heart failure; *HFrEF* HF with reduced ejection fraction, *HFmrEF* HF with mid-range ejection fraction,  *HFsemiq* HF with semiquantitatively estimated left ventricular ejection fraction - though <50%, *ESC* European Society of Cardiology, *RAS* renin-angiotensin system, *MRA* mineralocorticoid receptor antagonists | | | | | | | |
