# Supplementary material for: Differences in guideline-recommended heart failure medication between Dutch heart failure clinics: an analysis of the CHECK-HF registry
Source: Neth Heart J. 2020 May 19;28(6):334–44. doi: 10.1007/s12471-020-01421-1 (PMC7270463; doi:10.1007/s12471-020-01421-1)
Supplement: Supplementary file 7 — 7. Suppl. Table 7. Univariable analysis of predictors of HF medical treatment of HFrEF patients (LVEF <50%), Odds Ratios [95% confidence intervals] [file 12471_2020_1421_MOESM7_ESM.docx]

**Suppl. Table 7**. Univariable analysis of predictors of HF medical treatment of HFrEF patients (LVEF<50%),

Odds Ratios [95% confidence intervals]

|  | **Beta blocker** | **RAS inhibitor** | **MRA** | **Ivabradine** | **Diuretics** |
| --- | --- | --- | --- | --- | --- |
|  |  |  |  |  |  |
| Gender | 1.20 [1.07-1.35] | 0.79 [0.70-0.88] | 1.02 [0.93-1.11] | 1.24 [1.01-1.53] | 1.28 [1.13-1.45] |
| Age (per 10 years) | 0.87 [0.83-0.92] | 0.67 [0.64-0.71] | 0.93 [0.89-0.96] | 0.72 [0.67-0.78] | 1.42 [1.35-1.48] |
| BMI | 1.02 [1.01-1.03] | 1.03 [1.02-1.05] | 1.02 [1.01-1.03] | 1.02 [1.00-1.04] | 1.05 [1.04-1.06] |
| Systolic blood pressure (per 10 mmHg) | 1.00 [0.97-1.03] | 1.06 [1.03-1.09] | 0.82 [0.80-0.84] | 0.85 [0.80-0.90] | 0.83 [0.81-0.86] |
| Diastolic blood pressure (per 10 mmHg) | 1.05 [1.00-1.10] | 1.09 [1.04-1.15] | 0.82 [0.79-0.85] | 0.82 [0.75-0.89] | 0.74 [0.71-0.78] |
| NYHA classification | 0.92 [0.85-0.99] | 0.65 [0.59-0.70] | 1.35 [1.27-1.44] | 1.35 [1.16-1.56] | 2.26 [2.06-2.47] |
| Heart rate (per 10 beats/min) | 0.96 [0.92-0.99] | 0.84 [0.81-0.87] | 0.97 [0.94-1.00] | 1.00 [0.93-1.08] | 1.11 [1.06-1.16] |
| QRS duration (per 10 ms) | 0.97 [0.96-0.99] | 0.97 [0.96-0.99] | 1.04 [1.02-1.05] | 0.98 [0.94-1.01] | 1.07 [1.05-1.09] |
| eGFR (per 10 ml/min) | 1.02 [1.00-1.05] | 1.17 [1.14-1.21] | 1.00 [0.98-1.03] | 1.04 [0.99-1.09] | 0.81 [0.79-0.84] |
| Ischaemic aetiology | 1.00 [0.90-1.11] | 0.97 [0.86-1.09] | 0.99 [0.91-1.08] | 1.13 [0.91-1.40] | 1.08 [0.97-1.22] |
| Hypertension | 1.19 [1.05-1.35] | 1.09 [0.97-1.22] | 0.96 [0.87-1.05] | 0.76 [0.59-0.97] | 1.27 [1.11-1.45] |
| Diabetes | 1.07 [0.93-1.22] | 0.81 [0.71-0.92] | 0.99 [0.89-1.10] | 1.54 [1.21-1.95] | 1.57 [1.35-1.82] |
| COPD | 0.95 [0.82-1.11] | 0.75 [0.65-0.87] | 1.06 [0.94-1.19] | 1.45 [1.10-1.92] | 1.40 [1.19-1.66] |
| Renal insufficiency | 1.02 [0.90-1.14] | 0.49 [0.43-0.55] | 1.07 [0.98-1.17] | 0.82 [0.66-1.01] | 2.96 [2.60-3.36] |

† Defined as eGFR <60ml/min or a history of renal failure

*BMI* body mass index, *NYHA* New York Heart Association, *LVEF* left ventricular ejection fraction, *HF* heart failure, *HFrEF* HF with reduced ejection fraction, *RAS* renin-angiotensin system, *MRA* mineralocorticoid receptor antagonists, *BP* blood pressure, *eGFR* estimated glomerular filtration rate, *NT-proBNP* N-terminal pro-brain natriuretic peptide, *COPD* chronic obstructive pulmonary disease
