# Supplementary material for: Differences in guideline-recommended heart failure medication between Dutch heart failure clinics: an analysis of the CHECK-HF registry
Source: Neth Heart J. 2020 May 19;28(6):334–44. doi: 10.1007/s12471-020-01421-1 (PMC7270463; doi:10.1007/s12471-020-01421-1)
Supplement: Supplementary file 8 — 8. Suppl. Table 8. Practical recommendations for optimal use of guideline-directed heart failure therapies [file 12471_2020_1421_MOESM8_ESM.docx]

| \|  \| \| --- \|   **Suppl. Table 8.** Practical recommendations for optimal use of guideline-directed heart failure therapies | | | | |
| --- | --- | --- | --- | --- | --- |
| **At hospital level, providing:** | | |  | |
|  | Multidisciplinary team (MDT) care programme  Trained allied professionals (e.g. nurse-specialists and physician assistants)  Life-long learning programme for health-care professionals  Transmural collaboration | |  | |
|  | Up-to-date diagnostic and therapeutic protocols  Nurse-directed or pharmacist-directed optimisation of recommended medication  Advanced and palliative care programme  e-Health solutions, telemonitoring facilities  Benchmarking and adopting best practices  Monitoring of adherence, clinical outcomes and patient reported outcomes  Periodic review of performance, improving heart failure care accordingly | |  | |
| **At patient level, aimed at:** | |  | | |
|  | MDT is fully informed regarding medical history and social context  Assessing current condition and therapies  Management of comorbidities  Awareness of cognitive impairment and frailty  Matching patient profile and therapeutic options  Initiation, up-titration and maintenance of evidence-based therapies  Tailored dosing regimens and monitoring of blood pressure and heart rate  Addressing intolerabilities and side effects  Critical appraisal of polypharmacy  Patient education and counselling  Promoting self-management  Home-based monitoring, with implantable devices if applicable  In select patients: implantable pulmonary artery pressure monitoring  Addressing patient preferences and barriers  Involvement of family caregivers  Tailored end-of-life choices  Holistic approach preferably | | |  |
|  |  |  | | |
|  |  |  | | |
