# Supplementary figures and images for: Differences in guideline-recommended heart failure medication between Dutch heart failure clinics: an analysis of the CHECK-HF registry
Source: Neth Heart J. 2020 May 19;28(6):334–44. doi: 10.1007/s12471-020-01421-1 (PMC7270463; doi:10.1007/s12471-020-01421-1)

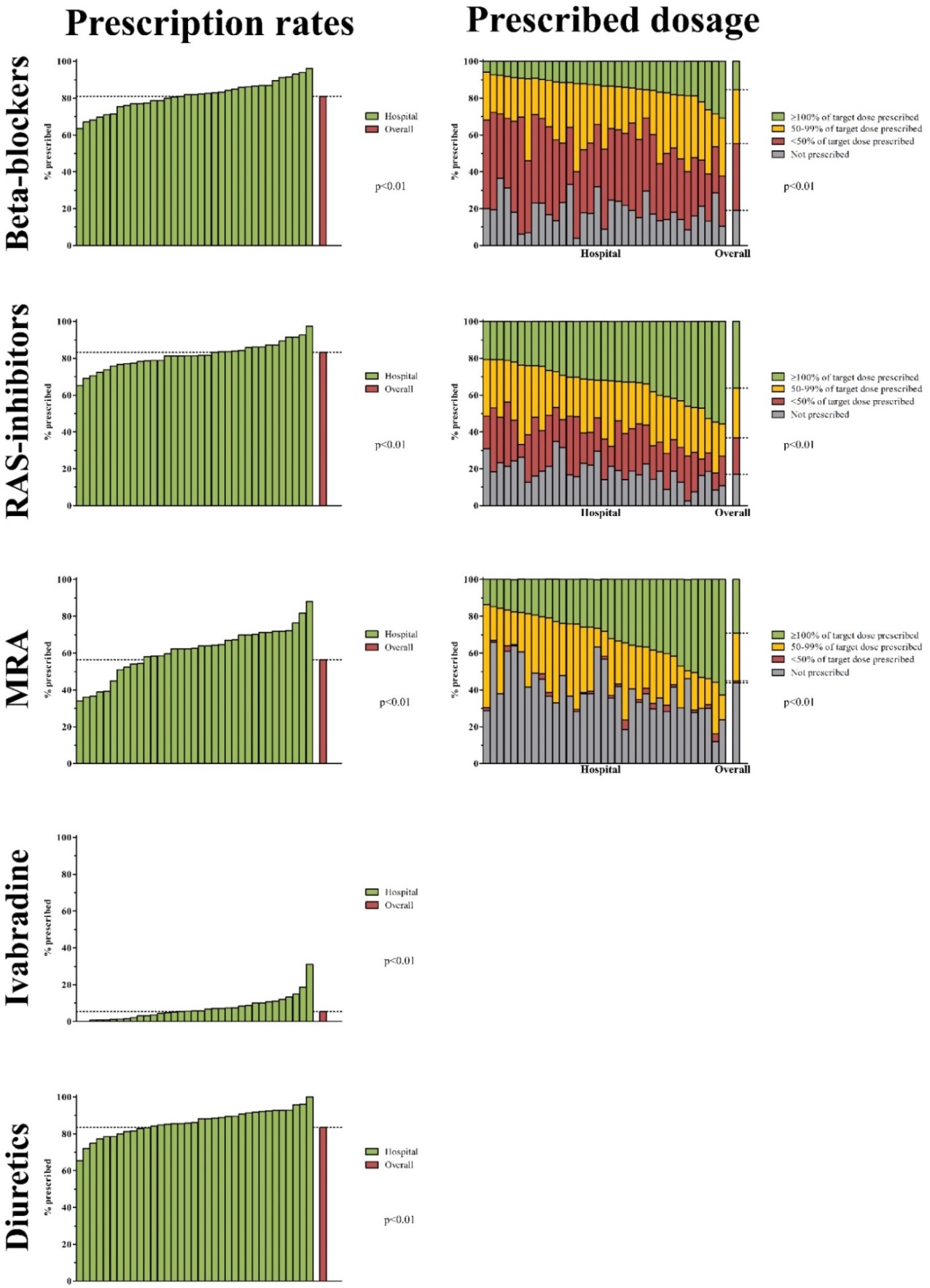

Supplement: Supplementary file 9 — 9. Suppl. Fig. 1. Prescription rates (%) and prescribed dosages (%) of HF medication in HFrEF patients (LVEF <40%) per participating clinic (n = 34) (The left panels show the order of hospitals on the x‑axis based on the percentage of prescription rate of each drug. The red bar is the overall prescription rate (%) and the green bars are the prescription rates (%) in each clinic. The same order is shown in the panels on the right). (HF heart failure, HFrEF heart failure with reduced ejection fraction, LVEF left ventricular ejection fraction, RAS renin-angiotensin system, MRA mineralocorticoid receptor antagonists) [file 12471_2020_1421_MOESM9_ESM.docx]

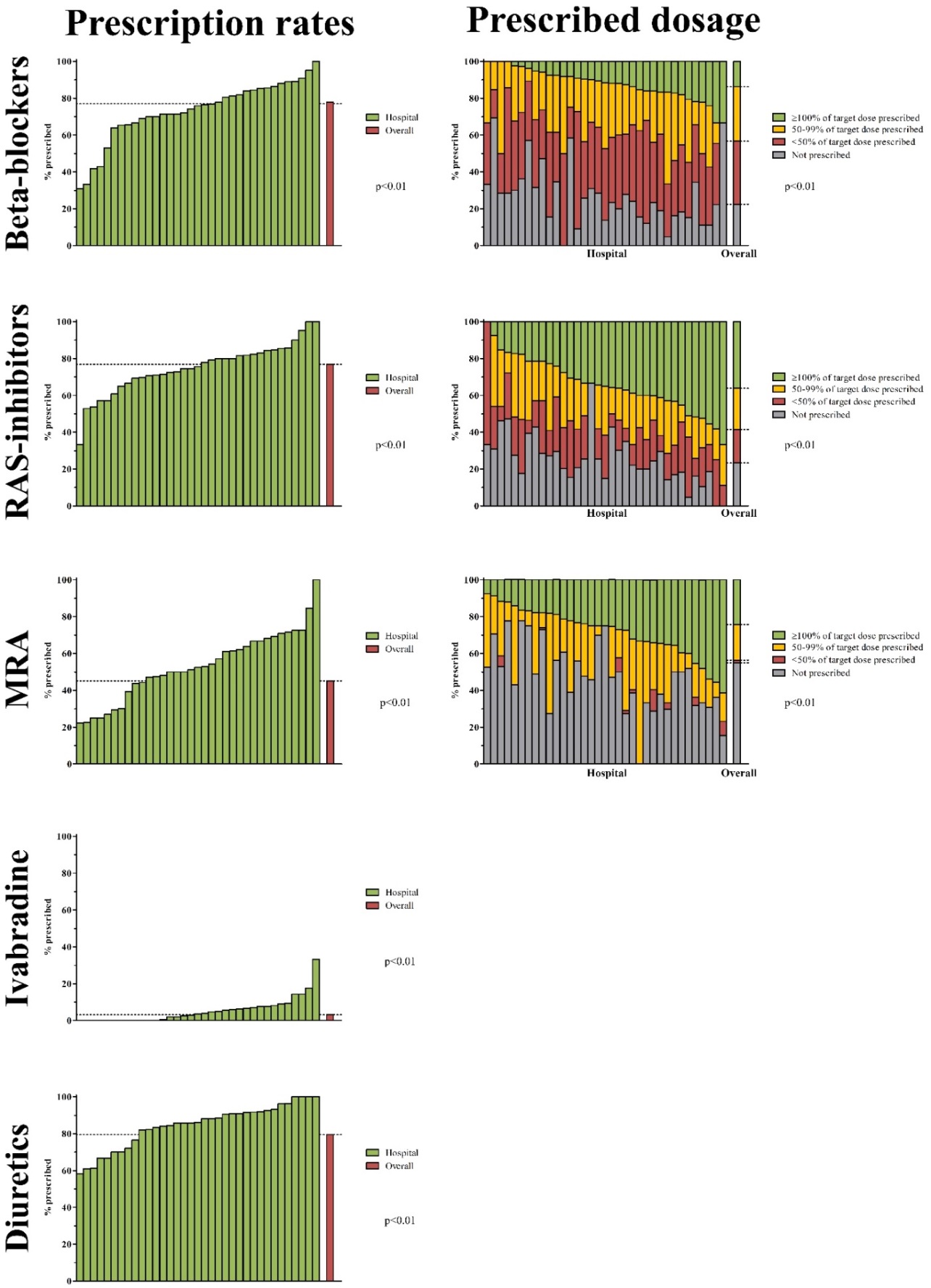

Supplement: Supplementary file 10 — 10. Suppl. Fig. 2. Prescription rates (%) and prescribed dosages (%) of HF medication in HFmrEF patients (LVEF 40–49%) per participating clinic (n = 34) (The left panels show the order of hospitals on the x‑axis based on the percentage of prescription rate of each drug. The red bar is the overall prescription rate (%) and the green bars are the prescription rates (%) in each clinic. The same order is shown in the panels on the right). (HF heart failure, HFmrEF heart failure with mid-range ejection fraction, LVEF left ventricular ejection fraction, RAS renin-angiotensin system, MRA mineralocorticoid receptor antagonists) [file 12471_2020_1421_MOESM10_ESM.docx]

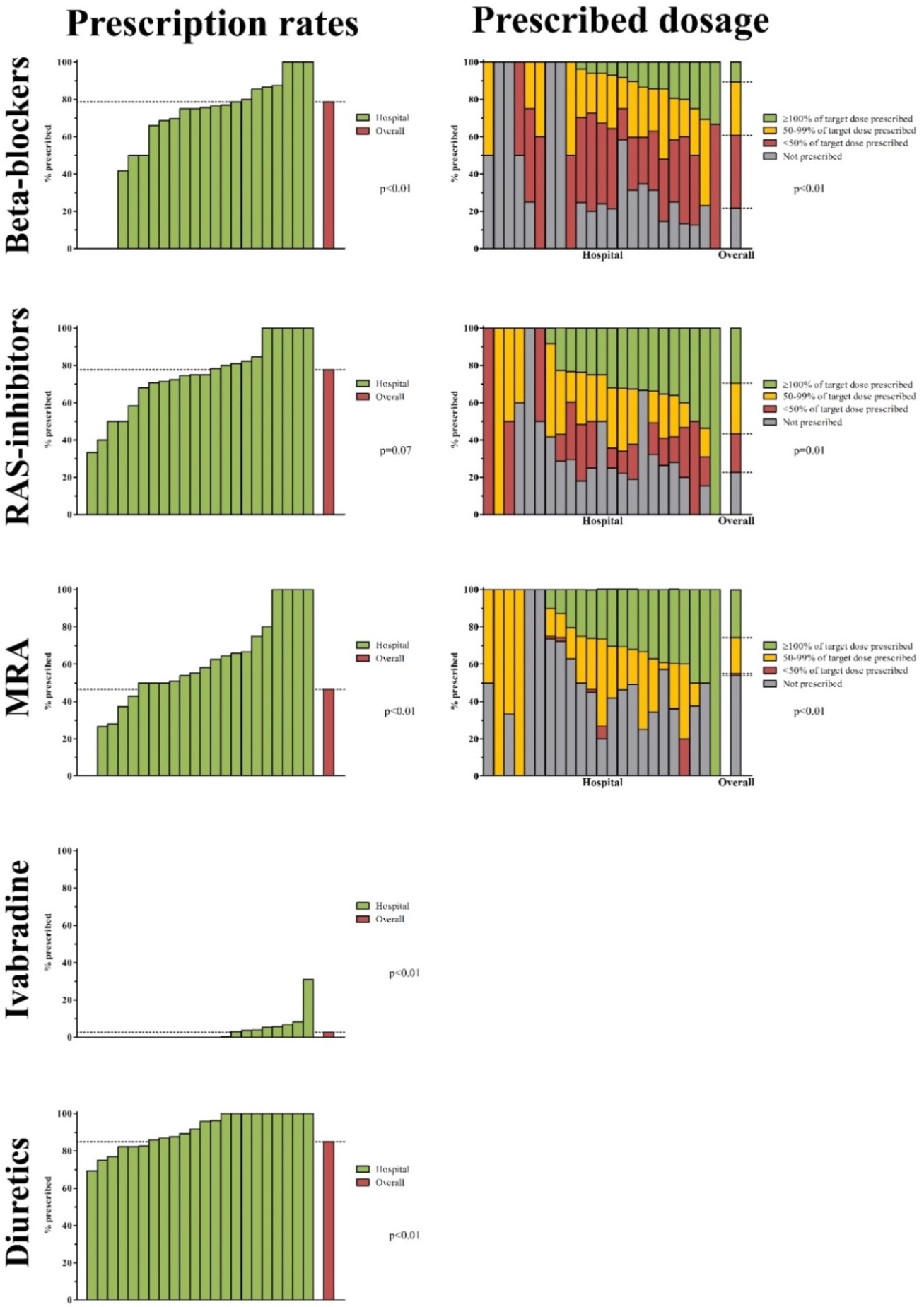

Supplement: Supplementary file 11 — 11. Suppl. Fig. 3. Prescription rates and prescribed dosages of HF medication in HF patients with semiquantitatively measured LV function per participating clinic (n = 27) (The left panels show the order of hospitals on the x‑axis based on the percentage of prescription rate of each drug. The red bar is the overall prescription rate (%) and the green bars are the prescription rates (%) in each clinic. The same order is shown in the panels on the right). (HF heart failure, LV left ventricular, RAS renin-angiotensin system, MRA mineralocorticoid receptor antagonists) [file 12471_2020_1421_MOESM11_ESM.docx]
